# Novel Mechanisms Underlying Rubber Accumulation and Programmed Cell Death in Laticiferous Canals of *Decaisnea insignis* Fruits: Cytological and Transcriptomic Analyses

**DOI:** 10.3390/plants12193497

**Published:** 2023-10-07

**Authors:** Yafu Zhou, Gen Li, Guijun Han, Shaoli Mao, Luyao Yang, Yanwen Wang

**Affiliations:** 1 Xi’an Botanical Garden of Shaanxi Province, Institute of Botany of Shaanxi Province, 17 Cui Hua Nan Road, Xi’an 710061, China; ligen@xab.ac.cn (G.L.); hgj@xab.ac.cn (G.H.);; 2 Shaanxi Engineering Research Centre for Conservation and Utilization of Botanical Resources, 17 Cui Hua Nan Road, Xi’an 710061, China

**Keywords:** programmed cell death, rubber biosynthesis, transcriptomic analysis, *Decaisnea insignis*, laticiferous canal

## Abstract

Natural rubber is one of the most important industrial raw materials, and its biosynthesis is still a fascinating process that is still largely unknown. In this research, we studied *Decaisnea insignis*, a unique rubber-producing plant that is different from other rubber-producing species due to the presence of lactiferous canals in its pericarp. The present study aims to provide novel insights into the mechanisms underlying rubber accumulation and PCD by subjecting the *Decaisnea insignis* laticiferous canals to light microscopy, TUNEL assay, and DAPI staining, as well as viability analysis, cellular ultrastructure analysis, and molecular analysis using light microscopy, scanning electron microscopy, immunofluorescence labeling, transmission electron microscopy, and transcriptome sequencing. At the cellular level, the origin of small rubber particles in the laticiferous canals had no morphological correlation with other organelles, and these particles were freely produced in the cytosol. The volume of the rubber particles increased at the sunken and expanding stage, which were identified as having the characteristics of programmed cell death (PCD); meanwhile, plenty of the rubber precursors or rubber particles were engulfed by the vacuoles, indicating a vacuole-mediated autophagy process. The accumulation of rubber particles occurred after the degeneration of protoplasts, suggesting a close association between rubber biosynthesis and PCD. The molecular analysis revealed the expression patterns of key genes involved in rubber biosynthesis. The upstream genes *DiIPP*, *DiFPP,* and *DiGGPPS* showed a decreasing trend during fruit ripening, while *DiHRT*, which is responsible for rubber particle extension, exhibited the highest expression level during the rubber particle formation. Moreover, the transcription factors related to PCD, *DiLSD1,* and *DiLOL2* showed a negative correlation with the expression pattern of *DiHRT*, thus exhibiting strict rules of sequential expression during rubber biosynthesis. Additionally, the expression trends of *DiXCP1* and *DiCEP1*, which act as proteases during PCD, were positively correlated with *DiGGPPS* expression. In conclusion, the findings suggest that the autophagic PCD may play a crucial role in rubber accumulation in *D. insignis*. Further research is still needed to fully understand the complex regulatory network underlying rubber biosynthesis in plants.

## 1. Introduction

Rubber is one of the most important industrial raw materials, which not only provides people access to everyday necessities, such as rubber goods for use in medicine and other light industries, but also provides numerous rubber products for manufacturing in other heavy industries. Rubber, or cis-1,4-polyisoprene, can be manufactured artificially or from the juice of various plants, and is therefore distinguished into synthetic (rubber) and natural rubber. The families of Moraceae, Asteraceae, Euphorbiaceae, Apocynaceae, Asclepiadaceae, Loranthaceae, Sapotaceae, Lardizabalaceae, and Aceraceae, among others, synthesize and accumulate natural rubber [1,2,3]. Excellent studies have been conducted on the biological synthesis of natural rubber due to its strategic importance.

The synthesis and accumulation of natural rubber has been known to form spherical rubber particles (RPs) in the secretory cells of plant secretory structures [4]. In the rubber tree (*Hevea brasiliensis*), there is a controversy regarding the synthesis of rubber particles. It is generally believed that the synthesis of rubber particles is related to the cytosol and organelles such as endoplasmic reticulum, vacuoles, and Golgi [2,4,5,6,7], although some researchers suggested that no morphological relationship exists between rubber synthesis and organelles [8]. In *Decaisnea insignis*, Hu et al. [9] studied the synthesis of rubber particles in the laticiferous canals, indicating a close relationship with endoplasmic reticulum and electron-dense small particles, while the formation of rubber particles in the vacuoles is related to the filamentous structure.

The biosynthesis of natural rubber occurs via a typical plant isoprenoid secondary metabolic pathway. In *Hevea brasiliensis*, the gene encoding a particle-bound rubber transferase (*HRT*), which is in charge of the cis-1,4-polymerization of isoprene units at the molecular level, was discovered by Asawatreratanakul et al. [10]. In a later study, Tang et al. [11] provided an explanation of the synthesis of natural rubber as following a typical plant isoprenoid secondary metabolic route and identified the key genes involved in rubber synthesis, including *IPP*, *FPP*, *REF*, *GGPPS,* and so on.

Globally, natural rubber is mainly obtained from the latex of *Hevea brasiliensis*; in addition, it exists in other plants such as *Taraxacum kok-saghyz*, *Parthenium hysterophorus*, *Parthenocissus tricuspidata*, *Ficus carica*, *Eucommia ulmoides*, and *Decaisnea insignis*, and its rubber particles are derived from the laticifer tubules, parenchyma, and laticiferous canals of the respective plant. A previous study by Zhou and Liu [12] reported that the origin and senescence of laticiferous canals in *Decaisnea insignis* is a developmentally regulated programmed cell death (PCD) process. Other studies have confirmed that the accumulation of secretions is accompanied by the degeneration of cellular components in plant glands [13,14,15,16,17]. The processes of ontogenesis in pigment glands of *Gossypium hirsutum* leaves [18]; secretory cavities of *Citrus sinensis* [19]; trichome cavities, secretory cavities, and capitate glandular hairs of *Dictamnus dasycarpus* [17,20,21]; and laticiferous canals of *Decaisnea insignis* [12] were studied, and the results showed that they were typical PCD events during which secretions accumulated. Zhao et al. [22] studied the proteomics of laticifer tubules in *E. kansui* and found that the degradation of cytoplasm was positively correlated with the synthesis of secondary metabolites during the development of lactiferous tubules.

Plants always die actively at the cellular, tissue, and organ levels to maintain normal growth and physiological metabolic activities [23,24]. Similar to animal apoptosis, PCD in plant is a complicated gene-regulated process that relies on the involvement of several proteases and transcription factors to carry out its essential functions. Caspases are one of proteases; they identify aspartic acid in the substrate and then cleave it off to degrade the intracellular matter. In plants, two proteins having caspase-like activities include CEP1 (Cysteine Proteinase Superfamily Protein) [25] and XCP1 (Xylem Cysteine Peptidase 1) [26], which have been demonstrated to be involved in the PCD process of Arabidopsis. The transcription factors LSD1 (lesion simulating disease1) [27] and LOL2 (lsd one like2) have also been discovered to negatively regulate the PCD process. In the plant glands, *CgCaN* and *CgPBA1* have been confirmed to be involved in DNA degradation during the PCD of *Citrus grandis* ‘Tomentosa’ secretory cavity cells [28,29]. Tong et al. [30] reported that *CisPG21* and *CisCEL16* are involved in cell wall degradation during the PCD of secretory cavities in the fruits of *C*. *sinensis*. However, few studies on the relationship between secretion, synthesis, and degeneration of secretory cells have been conducted in recent years at the molecular level.

The significance and function of the latex and rubber particles produced by *Decaisnea insignis* fruits remain a mystery. However, latex was supposed to have functions in defense and stress tolerance for higher expressed related genes compared with other tissues in *Hevea brasiliensis* [31]. Latex of *Decaisnea insignis* fruits contains 40% rubber hydrocarbon (cis-polyisoprene with molecular weight of 30–50 thousand) [9], which is similar to other natural rubbers. Laticiferous canal in fruits of *Decaisnea insignis* is big and concentrated; meanwhile, it is an extensive network in the pericarp of fruit, and rubber particles gradually accumulate in secretory epidermal cells at a certain developmental stage, which is different from those of other rubber plants, thus making it possible to follow the biological process of rubber synthesis. Our previous studies have confirmed that the development and senescence of laticiferous canals in *Decaisnea insignis* fruits is a programmed cell death (PCD) event [12], during which degenerated material originated from brown flocculent or irregular substance increases in size, and rubber accumulates; it is speculated that the biosynthesis of rubber particles is closely related to the cell degradation process of laticiferous canals. The present study aims to provide novel insights into the mechanisms underlying rubber accumulation and PCD by subjecting *Decaisnea insignis* laticiferous canals to light microscopy, TUNEL assay, and DAPI staining, as well as viability analysis, cellular ultrastructure analysis, and transcriptome sequencing using light microscopy, scanning electron microscopy, immunofluorescence labeling, and transmission electron microscopy.

## 2. Results

### 2.1. Development of Laticiferous Canal Formation and Rubber Accumulation

**Figure 1:**
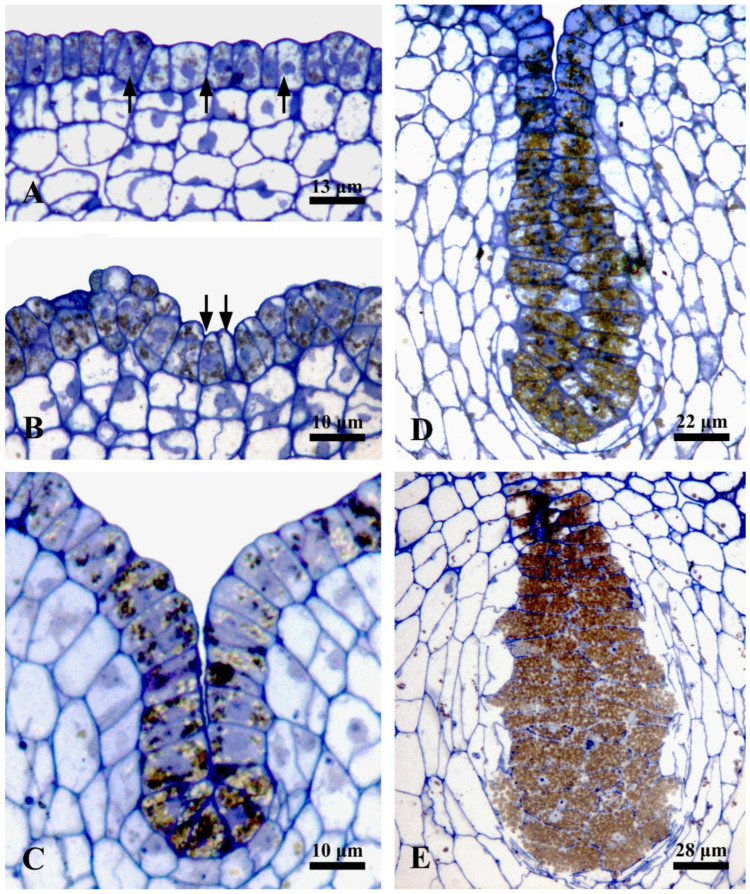
Development of laticiferous canal in *Decaisnea insignis* fruits. (**A**) Stage 1, earliest stage of the laticiferous canal identified by a layer of epidermal cells (arrows) with dense cytoplasm, evident nucleus, and a little amount of brown irregular flocculent substance. (**B**,**C**) Stage 2, sunken stage. (**B**) Early stage 2, an early sunken area (arrows) formed with increasing brown flocculent or irregular substance in the epidermal cells. (**C**) Stage 2, laticiferous canal characterized by an obvious sunken area with evident brown osmiophilic mass in sunken secretory epidermal cells. (**D**) Stage 3, plenty of brown rubber particles and a little amount of irregular flocculent substance in the secretory epidermal cells. (**E**) Late stage 3, showing deformed nuclei and rubber particles filled in the secretory epidermal cells.

**Figure 2:**
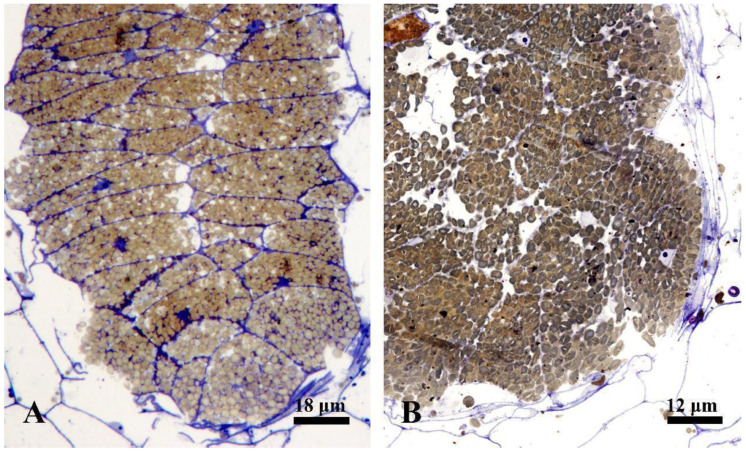
Late expanding stage and mature stage of laticiferous canal in *Decaisnea insignis* fruits. (**A**) Details of late expanding stage, deformed nuclei and rubber particles filled in the secretory epidermal cells. (**B**) Detail of mature stage, showing the secretory epidermal cells full of round or elliptical rubber particles, with thinner cell walls and disappearance of cytoplasm and nuclei.

**Figure 3:**
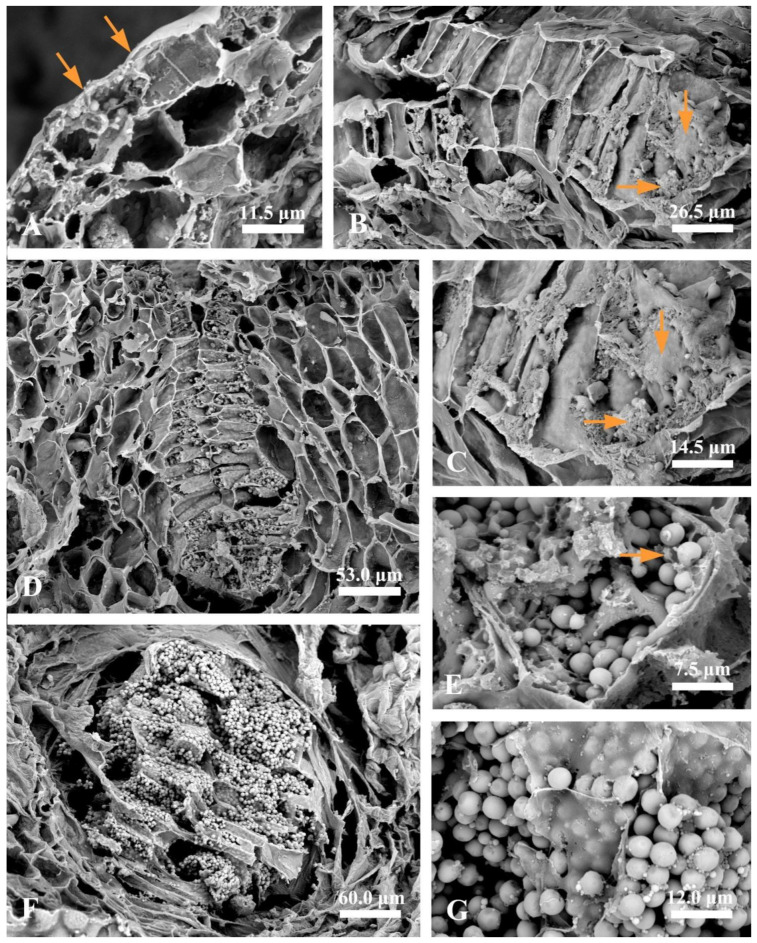
Rubber accumulation during the development of laticiferous canals in *Decaisnea insignis* fruits. (**A**) Earliest stage, no evident difference between the epidermis (arrows) and the ground meristem below the epidermis. (**B**) Late sunken stage, showing plenty of irregular substance (arrows) in the secretory epidermal cells. (**C**) Detail of irregular substance (arrows) in the secretory epidermal cells. (**D**) Expanding stage, plenty of rubber particles in the secretory epidermal cells, and no rubber particles exist in the adjacent parenchyma cells. (**E**) Details of rubber particles and a little amount of irregular substance (arrow) in the secretory epidermal cells. (**F**) Mature stage, the secretory epidermal cells filled with rubber particles. (**G**) Details of spherical rubber particles and thinner cell walls of the secretory epidermal cells.

The ontogenesis of laticiferous canals and the accumulation of rubber in the fruits of *Decaisnea insignis* were studied using the semi-thin section method. During the earliest stage, the laticiferous canals showed a layer of epidermal cells that were characterized by dense cytoplasm, evident nucleus, and a little amount of brown irregular flocculent substance, while no brown irregular flocculent substance was detected in the ground meristem below the epidermis (Figure 1A and Figure 3A). At the early stage 2, a sunken area was observed, which later developed into a laticiferous canal, and the amount of brown flocculent or irregular substance in the sunken secretory epidermal cells increased (Figure 1B). At a subsequent late sunken stage of rubber developmental phase, obvious brown osmiophilic mass was observed, especially in the bottom secretory epidermal cells of the laticiferous canals (Figure 1C and Figure 3B,C). At the later expanding stage, plenty of brown rubber particles were first observed, coupled with a little amount of irregular flocculent substance in the secretory epidermal cells, especially in the inner secretory epidermal cells (Figure 1D). The secretory epidermal cells were full of rubber particles, and most of the nuclei deformed or even degenerated during the late expanding stage (Figure 1E, Figure 2A, and Figure 3D,E). At the mature stage, the walls of the secretory epidermal cells were characterized by a thinner shape that was hard to recognize, and the secretory epidermal cells of the laticiferous canal were full of round or elliptical rubber particles (Figure 2B and Figure 3F,G).

**Figure 4:**
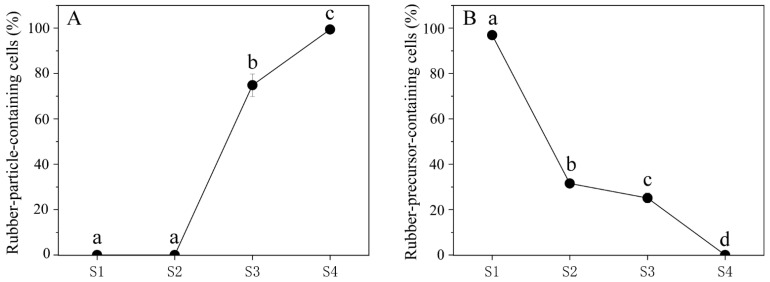
Percentage of rubber precursors and rubber particles in cells forming laticiferous canals of *Decaisnea insignis*. (**A**) Percentage of rubber particle-containing cells in laticiferous canals of *Decaisnea insignis* at different developmental stages. (**B**) Percentage of rubber precursor-containing cells in laticiferous canal of *Decaisnea insignis* at different developmental stages. Different letters on the bars indicate significant differences between different developmental stages.

To better understand the accumulation of rubber precursors (irregular osmiophilic flocculent or brown osmiophilic mass) and rubber particles during the development of laticiferous canals, we performed a statistical analysis of rubber precursors and rubber particles. The result showed that no rubber particles were observed in the secretory epidermal cells of the laticiferous canals at stage 1 and stage 2, but rubber particle-containing cells increased strikingly at stage 3 and stage 4 (Figure 4A). Rubber precursor-containing cells had the highest level at stage 1 (the earliest stage) and showed a steady decrease, and almost no rubber precursor-containing cells were observed at stage 4 (Figure 4B).

### 2.2. Cleavage of Nuclear DNA during Laticiferous Canal Formation

**Figure 5:**
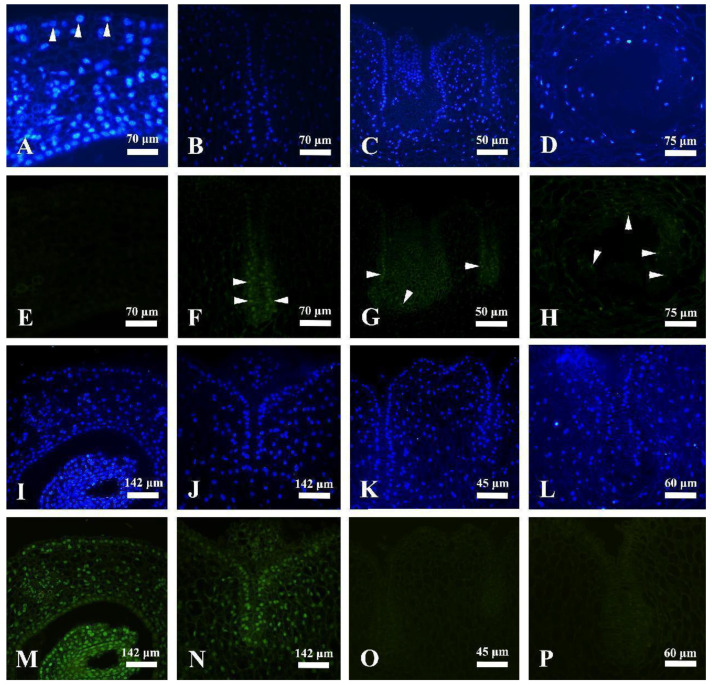
Detection of fragmented nuclear DNA and nucleic morphology using TUNEL assay and DAPI staining. (**A**) Earliest stage, showing evident nucleus with DAPI-positive reactions in secretory epidermal cells (arrowheads). (**B**) The sunken stage, some irregular nuclei observed with DAPI staining. (**C**) Expanding stage, most of the nuclei in the secretory epidermal cells of the laticiferous canal were completely degraded and were no longer visible. (**D**) The vast majority of nucleus degenerated and disappeared. (**E**) TUNEL-negative reaction in the earliest secretory epidermal cells. (**F**) The sunken stage, showing evident TUNEL-positive reaction in the secretory epidermal cells (arrowheads). (**G**) Expanding stage, most of the secretory cells were degraded and disappeared, the remaining secretory cells stained with DAPI were TUNEL-positive (arrowheads). (**H**) Mature stage, showing TUNEL-positive with weak fluorescence reaction in the remaining secretory epidermal cells (arrowheads). (**I**,**J**) DAPI-stained control of stage 1 and stage 2. (**K**,**L**) DAPI-stained control of stage 2 and stage 3. (**M**,**N**) TUNEL-positive control of stage 1 and stage 2, all the nucleus were showing TUNEL-positive reaction. (**O**,**P**) TUNEL-negative control of stage 2 and stage 3, all the nucleus were showing TUNEL-negative reaction.

Nuclear DNA degradation and nuclear changes during different developmental processes in laticiferous canals of *Decaisnea insignis* were detected using the TUNEL assay and DAPI staining. At the earliest stage, the secretory epidermal cells showed evident nucleus as indicated by a DAPI-positive reaction (Figure 5A) and a TUNEL-negative reaction (Figure 5E). At the sunken stage, the nuclei in the secretory epidermal cells were also stained as indicated by a DAPI-positive reaction (Figure 5B) and a TUNEL-positive reaction (Figure 5F), while the other adjacent parenchyma cells all showed a TUNEL-negative reaction (Figure 5F). At the expanding stage, we could see that most of the nuclei in the secretory epidermal cells were completely degraded, and the rest which was stained with DAPI stains (Figure 5C) showed a TUNEL-positive reaction (Figure 5G). At the mature stage, the remaining nuclei stained by DAPI (Figure 5D) in the secretory epidermal cells were TUNEL-positive with a weak fluorescence reaction (Figure 5H). The positive and negative control treatments were also conducted. At the earliest stage and sunken stage, when the DNase I treatment was conducted, all nuclei showing a DAPI-positive reaction in both the secretory epidermal cells and the adjacent parenchyma cells (Figure 5I,J) were TUNEL-positive (Figure 5M,N). In contrast, in the negative control, the nuclei in the adjacent parenchyma cells and the degenerated nuclei in the secretory epidermal cells at the sunken stage and the expanding stage (Figure 5K,L) were both TUNEL-negative (Figure 5O,P).

### 2.3. Viability

**Figure 6:**
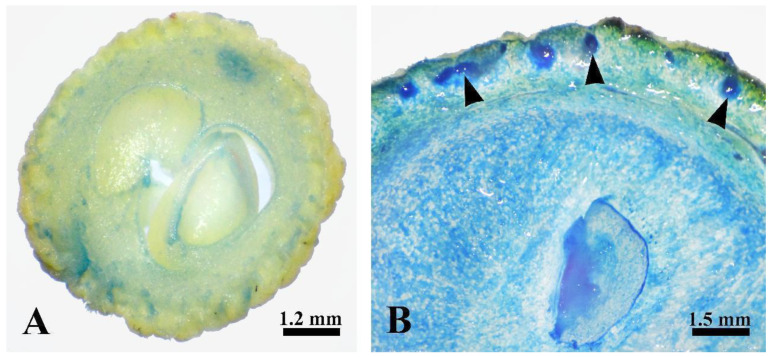
Viability of laticiferous canals in *Decaisnea insignis* pericarp stained with Evans blue stain. (**A**) Expanding stage, showing negative reaction of the laticiferous canals. (**B**) Mature stage, showing Evans blue-positive reaction of the laticiferous canals (arrowheads).

The developing fruits of *Decaisnea insignis* with laticiferous canals at the expanding stage and mature stage were stained using Evans blue to elucidate the viability of secretory epidermal cells. The results indicated that a negative reaction was detected during the expanding stage of the laticiferous canals (Figure 6A). At the mature stage, the laticiferous canals were evidently stained by the Evans blue stain, which could be distinguished from the other tissues in the pericarp (Figure 6B).

### 2.4. Rubber Accumulation and Nucleus Degeneration

**Figure 7:**
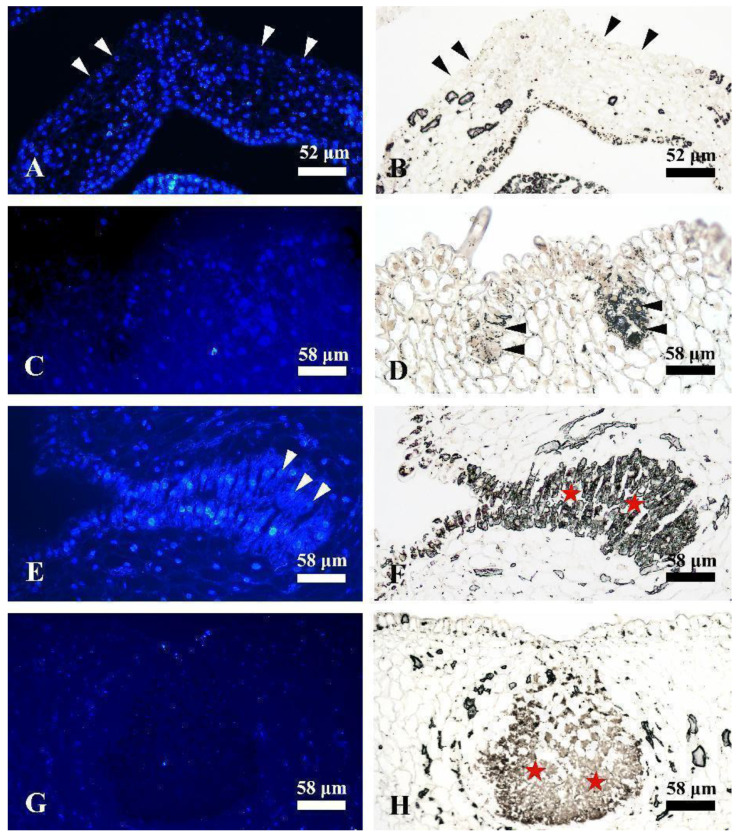
Rubber accumulation and nucleus degeneration. (**A**) Earliest stage, DAPI-positive nucleus of the secretory epidermal cells (arrowheads). (**B**) The earliest stage, showing no obvious rubber positive reaction in the secretory epidermal cells (arrowheads). (**C**) DAPI-positive nucleus of the secretory epidermal cells in early sunken stage. (**D**) Early sunken stage, showing obvious rubber accumulation in the secretory epidermal cells (arrowheads). (**E**) Expanding stage, showing the nuclei of secretory epidermal cells with evidently diffuse and deformed shape (arrowheads) or even disappeared completely. (**F**) Expanding stage, showing plenty of rubber accumulated in the secretory epidermal cells (asterisks). (**G**) DAPI-negative reaction in the nuclei of secretory epidermal cells. (**H**) Mature stage, showing the secretory epidermal cells (asterisks) filled with rubber particles.

Changes in rubber accumulation and nucleus degeneration in laticiferous canals of *Decaisnea insignis* at different developmental stages were examined using osmic acid and DAPI staining. The results showed that secretory epidermal cells were detected to have a DAPI-positive reaction (Figure 7A), while rubber particles showed a negative reaction at the earliest stage (Figure 7B). At the early sunken stage, DAPI-stained secretory epidermal cells (Figure 7C), with obvious blacker or high-intensity micro-dark osmiophilic substances, were detected (Figure 7D), especially in the bottom secretory epidermal cells (Figure 7D). At the expanding stage, the nuclei of the secretory epidermal cells were diffuse, deformed, or even disappeared completely (Figure 7E), while plenty of dark osmiophilic substances were observed in the secretory epidermal cells (Figure 7F). At the mature stage, the nuclei of the secretory epidermal cells were completely degraded and hard to observe (Figure 7G), while the secretory epidermal cells were filled with rubber particles (Figure 7H).

### 2.5. Ultrastructural Development of Laticiferous Canals

A cytological observation of rubber accumulation and degeneration of secretory epidermal cells at different developmental stages was conducted.

#### 2.5.1. Earliest Stage

**Figure 8:**
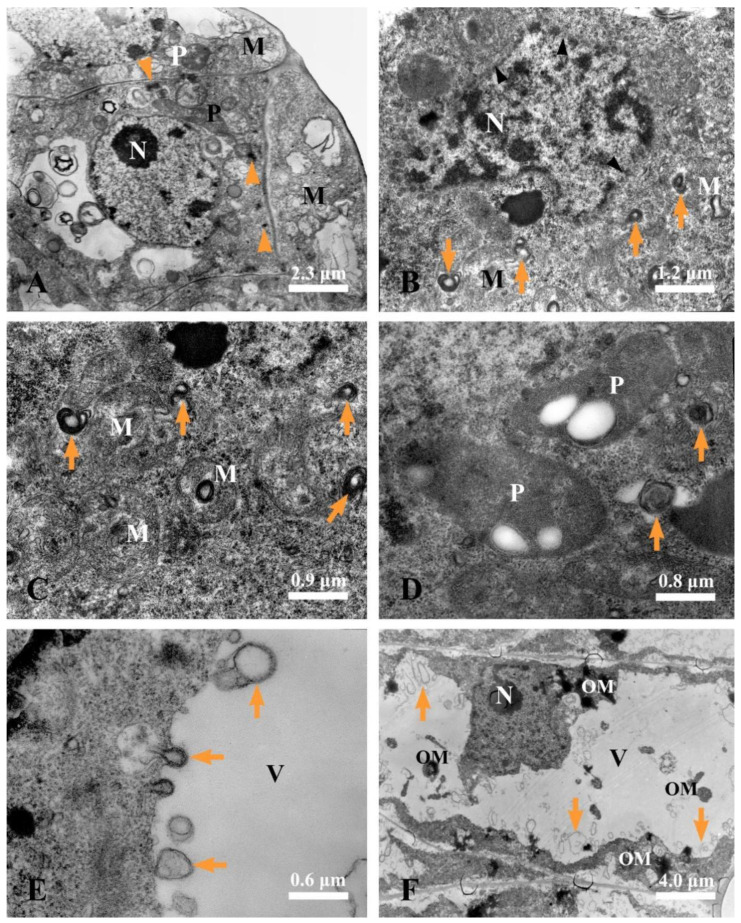
Ultrastructure of laticiferous canal in *Decaisnea insignis.* (**A**) Earliest stage, showing dense cytoplasm, large nucleus with evident nucleolus, abundant plastids and mitochondria, and dark osmiophilic substance (arrowheads) in the cytoplasm. (**B**) Sunken stage, showing condensed cytoplasm, deformed nucleus with condensed chromatin and diffused membrane (arrowheads) and membrane-like structures in the cytoplasm. (**C**) Sunken stage, deformed mitochondria with degenerated membrane and membrane-like structures (arrowheads) in the mitochondria and cytoplasm. (**D**) Membrane-like structures (arrowheads) in the cytoplasm and condensed plastids with fuzzy membrane. (**E**) Sunken stage, showing membrane-like structures (arrowheads) in the vacuole. (**F**) Sunken stage, showing membrane-like structures (arrowheads) and black osmiophilic mass in the vacuole and condensed cytoplasm. M, mitochondria; N, nucleus; OM, dark osmiophilic material; P, plastid; and V, vacuole.

At the earliest stage, the secretory epidermal cells were characterized by their dense cytoplasm, large nuclei, evident nucleoli, plastids, and mitochondria (Figure 8A). We could also observe flocculent material with a dark osmiophilic character in the cytoplasm (Figure 8A).

#### 2.5.2. Sunken Stage

As the development progressed, the secretory epidermal cells showed a condensed cytoplasm and a deformed nucleus with condensed chromatin and diffused membrane (Figure 8B), exhibiting early symptoms of degeneration. Plastids with degenerated membrane and condensed and deformed mitochondria were often found in the condensed cytoplasm (Figure 8C,D), and membrane-like structures were always observed close to the degenerated plastids, mitochondria, and nucleus (Figure 8B–D). At this stage, increasing membrane-like structures (Figure 8E,F) and black osmiophilic mass (Figure 8F) entered the vacuoles via vacuole endocytosis.

**Figure 9:**
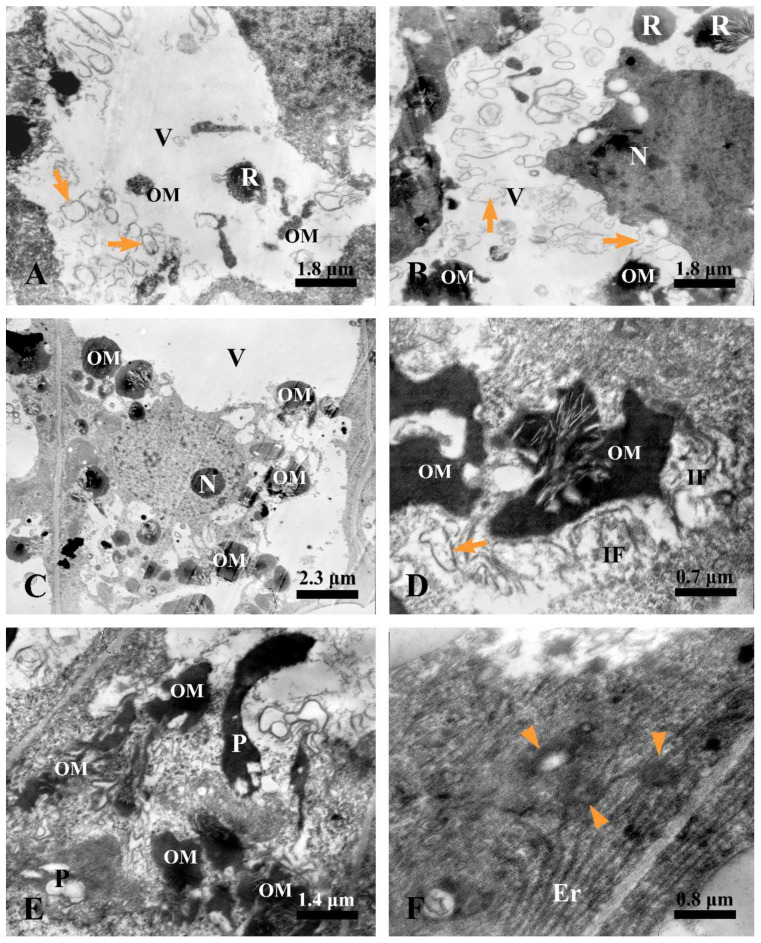
Ultrastructure of laticiferous canal in *Decaisnea insignis*. (**A**) Stage 2, showing more condensed cytoplasm of the secretory epidermal cells, plenty of membrane-like structures (arrows), and black osmiophilic mass in the vacuole. (**B**) Stage 2, showing membrane-like structures (arrows) and black osmiophilic mass in the vacuole and the degraded nucleus in the condensed cytoplasm. (**C**) Late stage 2, showing the degenerated nucleus pinching off into the vacuole, and black osmiophilic mass in the vacuole fused with the various substance from the cytoplasm. (**D**) Late stage 2, black osmiophilic mass in the vacuole fused with the various membrane-like structures (arrow) and irregular flocculent substance. (**E**) Late stage 2, showing strikingly condensed and disorganized cytoplasm, the plastids with condensed matrices, and black osmiophilic mass in the vacuole and disorganized cytoplasm. (**F**) Late stage 2, showing the abundance of endoplasmic reticulum with degraded ends (arrowheads) along the cell wall. Er, endoplasmic reticulum; IF, irregular flocculent substances; N, nucleus; OM, osmiophilic mass; P, plastid; R, rubber particle; and V, vacuole.

At the next stage, secretory epidermal cells with more condensed cytoplasm were detected. Meanwhile, we could observe plenty of membrane-like structures, irregular flocculent, and black osmiophilic mass in the vacuoles (Figure 9A,B). Then, the black osmiophilic mass in the vacuoles fused with various membrane-like structures and irregular flocculent substance from the cytoplasm, and its volume continued to increase during late stage 2 (Figure 9C,D), and the deformed nucleus was swallowed up by the vacuoles (Figure 9C). The cytoplasm at this stage was strikingly condensed and disorganized, the plastids showed a condensed matrix, and several endoplasmic reticula showed degraded ends along the cell wall (Figure 9E,F).

#### 2.5.3. Expanding Stage

**Figure 10:**
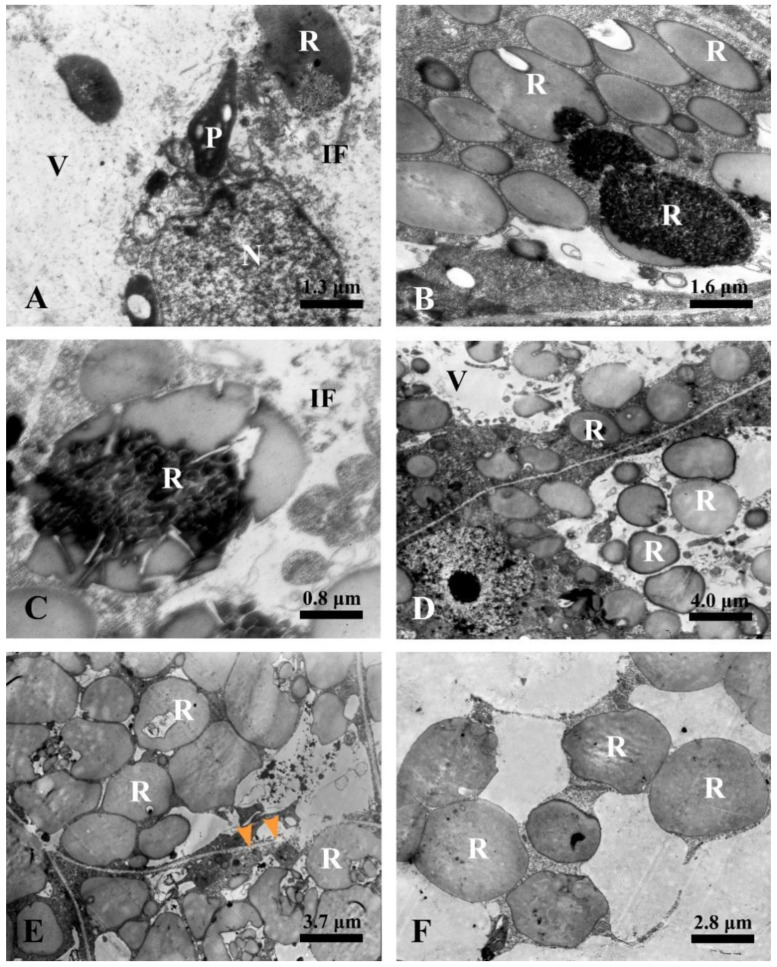
Ultrastructure of laticiferous canal in *Decaisnea insignis.* (**A**) Early stage 3, showing the degenerated plastid and nucleus in the vacuole, and various irregular flocculent substances in the vacuole combined on the rubber particles. (**B**) Stage 3, various substances merging with the rubber particles. (**C**) Stage 3, details of irregular flocculent substances combined with rubber particles in the vacuole. (**D**) Stage 3, showing plenty of rubber particles in the vacuole. (**E**) Mature stage, showing completely degraded protoplasts, thinner or degraded cell walls (arrowheads) of the secretory epidermal cells, and rubber particles with membrane-bound structure. (**F**) Details of rubber particles at maturity. IF, irregular flocculent substances; N, nucleus; P, plastid; R, rubber particle; and V, vacuole.

In the ultrastructures, the volume of the rubber particles increased in the secretory epidermal cells. The degenerated plastids and nuclei coupled with various degenerated substances were engulfed by the vacuoles (Figure 10A), and the irregular flocculent substances were constantly combined with the rubber particles (Figure 10A,B). Meanwhile, the secretory epidermal cells were distinguished by plenty of round rubber particles in the vacuole (Figure 10B–D).

#### 2.5.4. Mature Stage

At the mature stage, the secretory epidermal cells were characterized by completely degraded protoplasts and thinner or degraded cell walls (Figure 10E,F), and no intact vacuole was detected, and the cytoplasm and organelles were no longer visible. The laticiferous canals of *Decaisnea insignis* were filled with rubber particles that appeared to be membrane-bounded particles (Figure 10E,F).

### 2.6. RNA Sequencing, De Novo Assembly, and Functional Annotation

**Figure 11:**
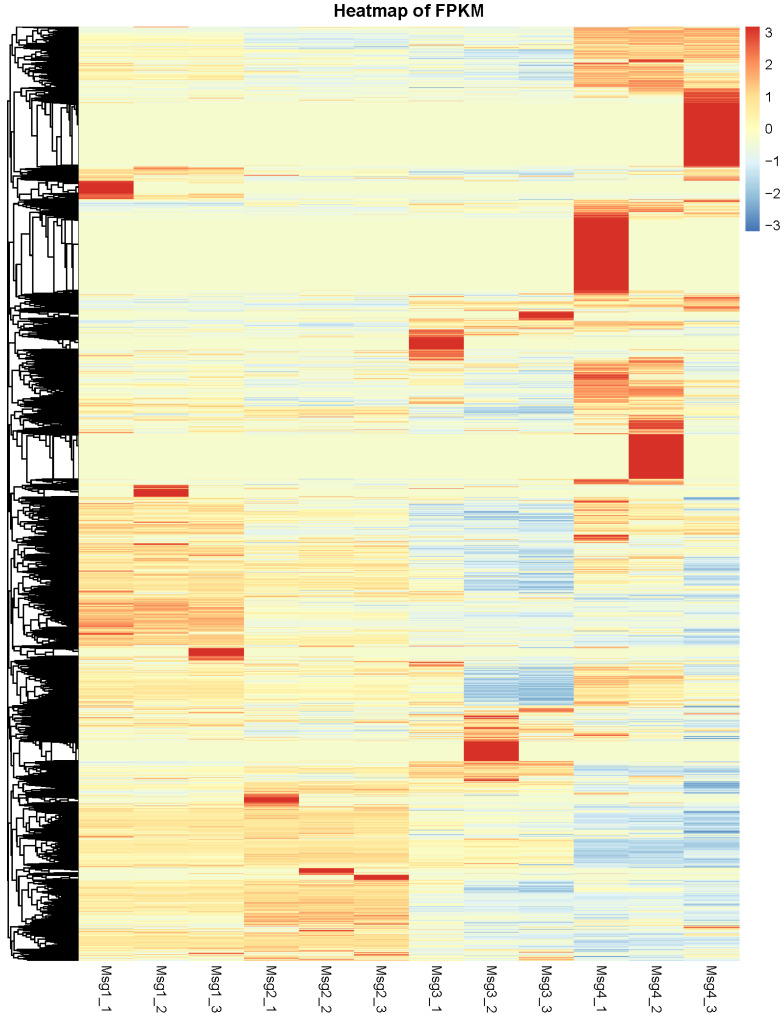
FPKM heatmap of laticiferous canals in *Decaisnea insignis* transcriptome during different developmental stages.

RNA sequencing of *Decaisnea insignis* fruit was performed for the first time in this study. Illumina HiSeq 2000 was used to generate and sequence 12 cDNA libraries based on the total RNAs of the fruit samples at different developmental stages during the fruit development change cycle. There were 307,145,894 raw reads in total, with 246,555,364 clean reads accounting for 80.27% of the total. A total of 67,944,392 clean reads were labeled as unique mapped reads, accounting for 27.56% of the total, while 178,610,972 were annotated as multiple mapped reads, accounting for 72.44% of the total. In addition, the clean reads had an average Q20, Q30, and GC content of 97.92%, 94.09%, and 44.69%, respectively. The transcripts and unigenes were functionally identified based on the excellent quality of the sequencing data. There were 218,020 transcripts found in total, with 38,802 (17.80%), 36,898 (16.92%), 43,986 (20.18%), 50,701 (23.26%), and 47,633 (21.85%) having lengthy intervals of 201–300 bp, 301–500 bp, 501–1000 bp, 1001–2000 bp, and >2000 bp, respectively. All transcripts had an average length of 1293.51 bp. Then, a total of 100,184 unigenes were annotated using the NR, GO, KEGG, eggNOG, Swiss-Prot, and Pfam databases, with 33,388 (33.33%), 25,863 (25.82%), 19,848 (19.81%), 11,339 (11.32%), and 9746 (9.73%) unigenes with lengths ranging from 200 to 301 bp and from 301 to 500 bp. All unigenes were 799.34 bp long on average. Among them, 38,853 unigenes were annotated to the NR database, accounting for 95.93 percent of all unigenes, while 10,192, 23,515, 12,383, 22,136, 21,109, and 20,729 unigenes were annotated to the COG, GO, KEGG, Pfam, KOG, and Swissport databases, respectively. These single genes were found to be spread throughout the three key Gene Ontology (GO) categories, including biological process (BP), cellular component (CC), and molecular function (MF), with 33,149, 23,881, and 34,199 unigenes in each group. FPKM was also employed as an indicator to assess the level of gene expression to reflect the expression level of transcripts (Figure 11).

### 2.7. Analysis and qRT-PCR Validation of Differentially Expressed Genes (DEGs)

**Figure 12:**
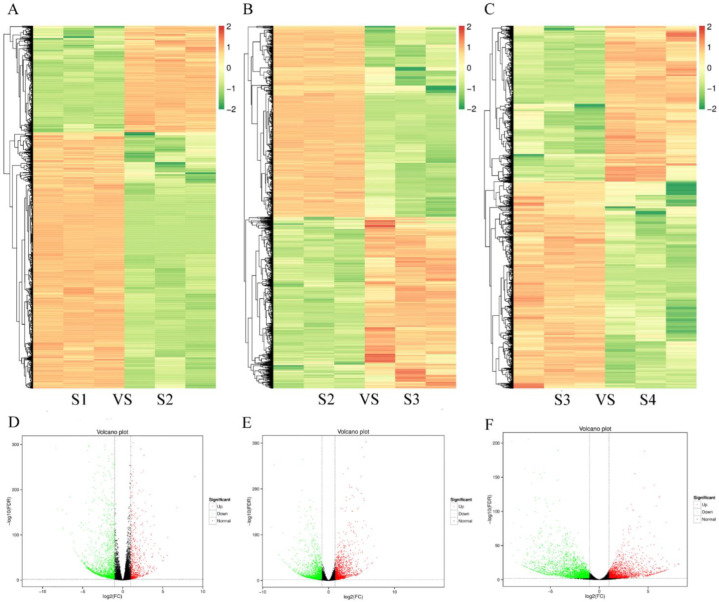
Comparison of DEGs in the development of laticiferous canals in *Decaisnea insignis.* (**A**) The heatmap of differentially expressed genes between stage 1 and stage 2. (**B**) The heatmap of differentially expressed genes between stage 2 and stage 3. (**C**) The heatmap of differentially expressed genes between stage 3 and stage 4. Reddish coloration indicates up-regulation of genes, and greenish color indicates a down-regulation of the gene. (**D**) The differential expressed genes between stage 1 and stage 2. (**E**) The differential expressed genes between stage 2 and stage 3. (**F**) The differential expressed genes between stage 3 and stage 4. *x*-axis represents the value of −log10 (padj); *y*-axis represents the value of log2 (fold change). Green spots represent down-regulated genes, and red spots represent the up-regulated genes.

To elucidate the genes involved in the physiological changes associated with different stages of *Decaisnea insignis* fruit development, we conducted an analysis of DEGs using the following criteria: *p*-value < 0.05 and |log2 FC (fold change)| ≥ 1. We identified a total of 2543 DEGs that exhibited differential expression across at least two developmental time points. Between stages S1 and S2, we discovered 747 up-regulated genes (represented by red dots) and 1796 down-regulated genes (represented by green dots) (Figure 12A,D). Similarly, between stages S2 and S3, we found 1645 up-regulated genes and 1827 down-regulated genes (Figure 12B,E). Additionally, between stages S3 and S4, we identified 2035 up-regulated genes and 2704 down-regulated genes (Figure 12C,F). From these DEGs, we focused on genes associated with the regulation of programmed cell death and the synthesis of natural rubber. To validate the accuracy and reliability of our transcriptome data, we performed qRT-PCR to measure the expression levels of DEGs involved in cellular autophagy and the natural rubber biosynthesis pathway across stages S1 to S4 (Figure 12). The relative expression patterns of these candidate genes were consistent with the results obtained from the RNA-seq, thus confirming the precision and dependability of our transcriptome data.

**Figure 13:**
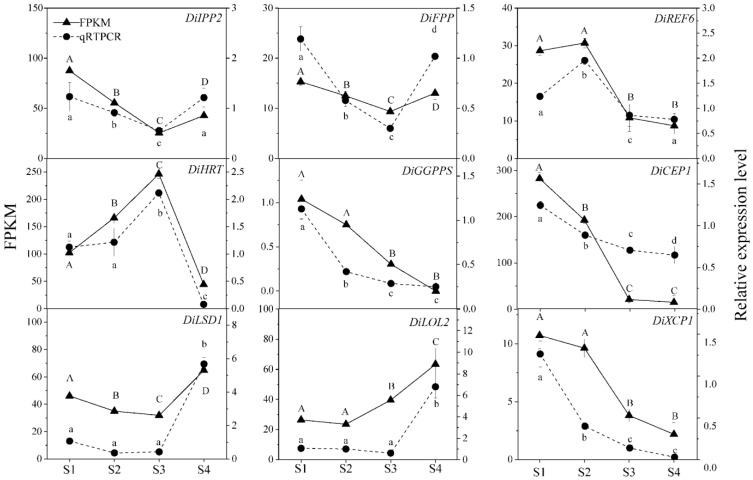
Changes in qPCR expression and FPKM value of transcriptome during the development of laticiferous canals in *Decaisnea insignis* (*p* < 0.05). Transcript levels of *DiIPP2*, *DiFPP*, *DiREF6*, *DiHRT*, and *DiGGPPS*, belonging to the natural rubber synthesis pathway genes, and transcript levels of *DiLSD1*, *DiCEP1*, *DiXCP1,* and *DiLOL2*, belonging to the PCD-related gene. The data show the means ± SDs (*n* = 3). Different letters on the bars indicate significant differences between different developmental stages.

To investigate the relationship between the rubber synthesis pathway and programmed cell death (PCD), we further examined the transcript abundance of the selected genes using qPT-PCR (Figure 13). During the development of laticiferous canals in *Decaisnea insignis* fruits, the expression levels of *DiIPP2* and *DiFPP*, which were involved in rubber synthesis, exhibited a significant decreasing trend from stages S1 to S3, followed by a rebound in expression during the S4 stage. Conversely, the key gene *DiHRT* displayed an increasing trend in expression from stages S1 to S3 and a significant decrease in expression during stage S4. Additionally, the expression level of *DiGGPPS* decreased from S1 to S4, whereas *DiREF6* showed higher expression levels during S1 to S2 and lower expression levels in S3 and S4. Similarly, PCD-related genes encoding proteases and transcription factors also showed distinct patterns at different developmental stages. Both *DiCEP1* and *DiXCP1* exhibited a decreasing trend in expression as the fruits developed. Conversely, the PCD negatively regulated transcription factors, *DiLSD1* and *DiLOL2*, which displayed low expression levels during S1 to S3 but showed a significant increase in expression at the mature stage.

To further assess the accuracy and dependability of the transcriptome data, qRT-PCR was used to measure the expression levels in S1-S4 DEGs associated with cellular degeneration and the pathway leading to the production of natural rubber (Figure 13). These candidate genes’ relative expression patterns matched those obtained using RNA-seq, thus proving the accuracy and dependability of the transcriptome data.

## 3. Materials and Methods

### 3.1. Plant Materials

**Figure 14:**
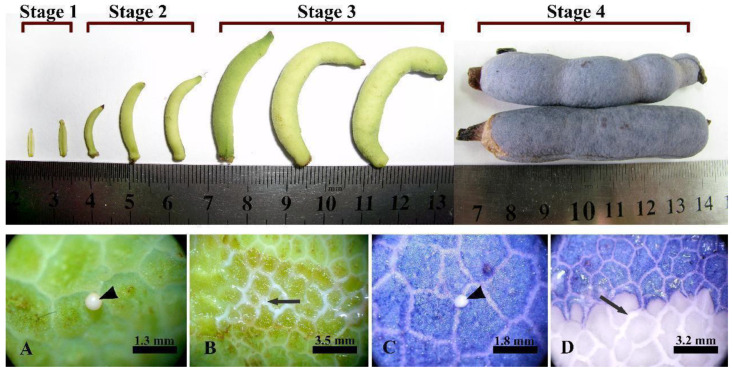
Development of *Decaisnea insignis* fruits and latex accumulation. Stage 1, earliest stage; stage 2, sunken stage; stage 3, expanding stage; and stage 4, mature stage. (**A**,**B**) Late expanding period, showing latex (arrowhead) and reticular laticiferous canal (arrow) on the surface of the fruit. (**C**,**D**) Maturity, latex (arrowhead) and reticular laticiferous canal (arrow) on the surface of the fruit. Arrowhead showing latex flowing out after acupuncture at the outer area of the laticiferous canal on the pericarp; arrow, showing the laticiferous canal under the outer pericarp after the pericarp being tangentially cut.

*Decaisnea insignis* (Griffith) J. D. Hooker et Thomson is a perennial shrub. The samples of *Decaisnea insignis* fruits were collected from Qinling Mountains, Shannxi, China (33.54858073N, 108.366563E). The developmental process of laticiferous canals in *Decaisnea insignis* were subdivided into four stages according to our previous study [12]: earliest, stage 1 (S1); sunken, stage 2 (S2); expanding, stage 3 (S3); and mature, stage 4 (S4) (Figure 14).

### 3.2. Light Microscopy

The fruit samples of *Decaisnea insignis* at different developmental stages were divided into small pieces of about 1 mm^3^. The samples were first pre-fixed with 2.5% glutaraldehyde in 0.1 M phosphate buffer at a pH of 7.0 for 48 h at 4 °C, and then fixed with 1% osmic acid in the same phosphate buffer overnight. After rinsing with the phosphate buffer three times (30 min each time), the samples were dehydrated in an ethanolic series (30%, 50%, 70%, 85%, and 95% one time, and 100% twice, for 30 min each time), prepared with propylene oxide, and finally embedded in Epon 812 resin [21]. Semi-thin sections (1–2 μm) were obtained using a Reichert–Jung ultramicrotome, and then stained with toluidine blue O or methylene blue. A Leica microscope (DMLB) equipped with a video camera (DFC 7000T; Wetzlar, Germany) was used to examine the sections, and the data obtained from the examination were recorded.

### 3.3. Scanning Electron Microscopy

Samples of *Decaisnea insignis* fruits at different developmental stages were prepared by pre-fixing them with 2.5% glutaraldehyde in 0.1 M phosphate buffer (pH 7.0) for 48 h, and post-fixing in 1% osmium tetroxide overnight at 4 °C. After dehydration using a graded ethanolic series, the samples were treated with tertiary butyl alcohol two times, and then placed under −20 ℃ overnight and vacuumed for 24 h in succession. The dried samples were then mounted on stubs, coated with gold for 1 min using a Hitachi E-102 Ion sputter (Hitachi High-Technologies Corporation, Tokyo, Japan), and observed using a Hitachi TM-1000 tabletop scanning electron microscope (Hitachi, Tokyo, Japan).

### 3.4. Evans Blue

The cross sections of the *Decaisnea insignis* fruit samples at stage 2 and stage 4 were first sliced using the hand sectioning technique and then stained with 0.1% Evans blue dissolved in ddH_2_O for 30 min at room temperature [21]. Finally, the samples were washed with distilled water and photographed using a JSZ5BS light microscope (Jiangnan, China) equipped with a Nikon D850 (Nikon, Japan).

### 3.5. Osmic Acid and DAPI Staining

Rubber could be stained using osmic acid [32]. To reflect the relationship between rubber accumulation and nuclear degeneration, the samples at different developmental stages were divided into small pieces of about 0.5 mm^3^. The samples were first pre-fixed with 2.5% glutaraldehyde in 0.1 M phosphate buffer at pH 7.0 for 48 h at 4 °C, and then fixed with 1% osmic acid using the same phosphate buffer overnight. After rinsing with the phosphate buffer three times (30 min each time), the samples were dehydrated using a gradient ethanolic series (30%, 50%, 70%, 85%, and 95% one time, and 100% twice, for 30 min each time), prepared using xylene, and finally embedded in wax [21]. For rubber particle and nucleus locating, 8–10 μm sections of different stages of *Decaisnea insignis* at different stages were obtained and stained with 2 mg of DAPI in 10 mL of dilution buffer (Bioworld Technology, Nanjing, China), and observed using an epifluorescence microscope. The nuclei and RPs were observed using an excitation wavelength of 340–380 nm and under bright field using a Leica DMLB epifluorescence microscope.

### 3.6. TUNEL Assay and DAPI Staining

To detect DNA fragmentation, the TUNEL procedure was applied using a TUNEL (terminal deoxynucleotidyl transferase-mediated dUTP nick-end labeling reaction) apoptosis detection kit (KeyGen Biotech, Nanjing, China). After the samples were embedded in wax, 8 μm sections were created using a Leica RM 2135 rotary microtome; then, the dewaxed samples were rinsed with 0.1 mol L^−1^ phosphate buffer (1% BSA) at a pH of 7.4. The samples were then made permeable by incubating the sections with 100 μL of Proteinase K (10 μL 10× Proteinase K in 90 μL of 0.1 M phosphate buffer, pH 7.4) in a humid chamber for 30 min at 37 °C, followed by three rinses with phosphate-buffered saline. DNA fragmentation was detected directly according to the manufacturer’s instructions for 1 h at 37 °C in total darkness using a TUNEL apoptosis detection kit (KeyGen Biotech, Nanjing, China). For each experiment, one positive control was incubated with 100 μL of DNase I (50 U·μL^−1^) for 30 min at 37 °C before labeling, and a negative control was included in each experiment by omitting the terminal deoxynucleotidyl transferase (TdT) enzyme. Furthermore, the TUNEL-labeled sections were stained in total darkness for 30 min at 37 °C, and then stained with 2 mg of DAPI in 10 mL of dilution buffer (Bioworld Technology, Nanjing, China).

Finally, the stained sections were observed at 450–500 nm and 340–380 nm using a Leica epifluorescence microscope (DMLB) equipped with a video camera (DFC 7000T; Wetzlar, Germany) for TUNEL and DAPI detection, respectively.

### 3.7. Transmission Electron Microscopy

For ultrastructure analysis, small fragments of the *Decaisnea insignis* fruit samples were fixed, dehydrated, and embedded in Epon 812; thin sections (60–80 nm) were cut using a Leica EM UC 6 ultramicrotome and then stained with uranyl acetate and lead citrate [21], and examined using an H-600 TEM (Hitachi, Japan).

### 3.8. RNA Library Preparation for Transcriptome Sequencing

To assure the use of certified samples for transcriptome sequencing, the purity, concentration, and integrity of the RNA samples were checked spectrophotometrically using 1% (*w*/*v*) agarose gel electrophoresis. To prepare the RNA samples, a total of 1 μg of RNA per sample was used as the input material. Following the manufacturer’s guidelines, sequencing libraries were created using the NEB Next^®^ UltraTM RNA Library Prep Kit for Illumina^®^ (NEB, Ipswich, MA, USA), and index codes were added to assign sequences to each sample. Using poly-T oligo-attached magnetic beads, mRNA was extracted from total RNA. In NEB Next First-Strand Synthesis Reaction Buffer (5X), fragmentation was carried out by utilizing divalent cations at a high temperature. M-MuLV reverse transcriptase and a random hexamer primer were used to make first-strand cDNA. Following that, DNA polymerase I and RNase H were used to synthesize second-strand cDNA. Exonuclease/polymerase activities were used to convert the remaining overhangs into blunt ends. To prepare for hybridization, NEB Next Adaptor with a hairpin loop structure was ligated after adenylation of the 3’ ends of DNA fragments. The library fragments were purified using the AMPure XP technology to select cDNA fragments with a length preference of 240 bp (Beckman Coulter, Beverly, NJ, USA). Then, before PCR, 3 μL of USER Enzyme (NEB, Ipswich, MA, USA) was added to size-selected, adaptor-ligated cDNA for 15 min at 37 °C, followed by 5 min at 95 °C. Then, using Phusion high-fidelity DNA polymerase, universal PCR primers, and index (X) primer, PCR was carried out. Finally, the PCR products were purified (using the AMPure XP system), and the quality of the library was determined using the Agilent Bioanalyzer 2100 system. The index-coded samples were clustered using the TruSeq PE Cluster Kit v3-cBot-HS (Illumia) via a cBot Cluster Generation System according to the manufacturer’s instructions. The library preparations were sequenced using the Illumina Hiseq 2000 platform and paired-end reads were generated after cluster creation.

### 3.9. Transcriptome Assembly, Gene Functional Annotation, and Quantification of Gene Expression Levels

All left files (read1 files) from all libraries/samples were combined into a single large left.fq file, and all right files (read2 files) were combined into a single large right.fq file. Trinity [33] was used to assemble the transcriptome based on the left.fq and right.fq files, with the min kmer cov set parameter to 2 by default and all other parameters set as default. The following databases were used to annotate gene function: NR (NCBI non-redundant protein sequences); Pfam (protein family); KOG/COG/eggNOG (clusters of orthologous groups of proteins); Swiss-Prot (a manually annotated and reviewed protein sequence database); KEGG (Kyoto Encyclopedia of Genes and Genomes); and GO (Gene Ontology). For each sample, RSEM [34] was used to evaluate gene expression levels, and the clean data were remapped onto the assembled transcriptome, and the read count for each gene was calculated using the mapping findings.

### 3.10. Differential Gene Expression Analysis

The DESeq R package (1.10.1) was used to perform differential expression analysis of two groups. Using a model based on a negative binomial distribution, DESeq provides statistical methods for identifying differential expression in digital gene expression data. The false discovery rate was controlled by adjusting the *p*-values using the Benjamini–Hochberg’s method. Genes discovered by DESeq with an adjusted *p*-value of 0.05 were labeled as differentially expressed.

### 3.11. Functional Enrichment Analysis of Differentially Expressed Genes (DEGs)

The topGO R package based on the Kolmogorov–Smirnov test was used to conduct a Gene Ontology (GO) enrichment analysis of differentially expressed genes (DEGs). KEGG [35] is a database resource for deducing high-level functions and utilities of biological systems, such as cells, organisms, and ecosystems, from molecular-level data, particularly large-scale molecular datasets generated via genome sequencing and other high-throughput experimental technologies (http://www.genome.jp/kegg/, accessed on 13 October 2020). To examine the statistical enrichment of differentially expressed genes in the KEGG pathways, we employed the KOBAS software [36].

### 3.12. Quantitative Reverse Transcription-PCR (qRT-PCR)

The total RNA from the samples was extracted using an RNA prep Pure Plant Kit (DP432, TianGen, Beijing, China), and the cDNA was synthesized via reverse transcription using a Fasting cDNA First-Strand Synthesis Kit (TianGen, Beijing, China) according to the manufacturer’s instructions. For the relative gene expression assay, the housekeeping gene β-actin was employed as an internal control, as it is assumed to exhibit a uniform expression in *Decaisnea insignis*. The qRT-PCR procedure was established using a LightCycler96 Real-Time PCR Detection System (Roche, Switzerland) with a qRT-PCR kit (Roche, Switzerland) according to the manufacturer’s recommendations. Each 20 μL reaction contained 10 μL of 2× FastStart Essential DNA Green Master Mix (SYBR Green), 1 μL of forward primer (10 μM), 1 μL of reverse primer (10 μM), 1 μL of cDNA template (20 ng), and 7 μL of H_2_O. The following conditions for qRT-PCR were designed and tested in a three-step assay: 95 °C/10 min (one cycle), 95 °C/10 s, 55 °C/10 s, and 72 °C/20 s (40 cycles). The data were analyzed using the 2^–ΔΔCt^ method [37,38]. The relative expression levels are expressed as the mean ± SD of three replicates. Parameter differences among the four developmental stages of gene expression parameters across the four developmental stages were determined using one-way ANOVA with appropriate post hoc analysis (*p* < 0.05). The line figures were drawn using Origin 9.0. The primers for qRT-PCR are shown in Supplementary Table S1.

### 3.13. Percentage of Rubber Precursor and Rubber Particle-Containing Cells

The semi-thin sections of laticiferous canals of *Decaisnea insignis* fruits were observed and counted using a 20× microscope, with three biological replicates for each developmental period; the total number of secretory epidermal cells, the number of rubber precursors (irregular osmiophilic flocculent or brown osmiophilic mass) containing secretory epidermal cells, and the number of secretory epidermal cells containing rubber particles were counted. The presence of irregular osmiophilic flocculent and black osmiophilic mass (instead of rubber particles) was counted in the number of rubber precursor-containing cells; cells containing only rubber particles were counted in the number of rubber particle-containing cells. And the proportion of target cells to total cells was calculated as follows: percentage of target cells (%) = number of target cells/total number of secretory epidermal cells × 100%. Parameter differences among the four developmental stages of laticiferous canal cells were determined using one-way ANOVA with appropriate post hoc analysis (*p* < 0.05). The line figures were drawn using Origin 9.0.

## 4. Discussion

### 4.1. Special Material for Natural Rubber Biosynthesis Study

Presently, natural rubber is known to be produced in latex vessel systems or laticifers of the *Hevea brasiliensis* [31], while in guayule, rubber particles are predominantly biosynthesized and accumulated in the epithelial parenchyma cells lining the resin ducts of the stem [39]. *Decaisnea insignis* is a perennial shrub in Lardizabalaceae family, the pericarp of which is characterized by lactiferous canals in which rubber particles accumulate, and no lactiferous canal or laticifer is distributed in the other organs [12,40]; furthermore, laticiferous canal in fruits of *Decaisnea insignis* is big and concentrated; meanwhile, it is an extensive network in the pericarp of fruit, and rubber particles gradually accumulate in secretory epidermal cells at a certain developmental stage. Taken together, *Decaisnea insignis* is different from lactiferous plants or other rubber plants such as *Hevea brasiliensis* and *Parthenium argentatum*. It is very helpful to study the biological synthesis process of natural rubber during the development of laticiferous canals.

### 4.2. Freely Produced Rubber Precursors in Cytoplasm

At the cellular level, the biological synthesis of natural rubber has remained controversial, although a great number of research studies have been conducted. Hu et al. [9] studied the formation of rubber particles in the laticiferous canals of *Decaisnea insignis*, showing that the formation of rubber particles is closely related to the endoplasmic reticulum and electron-dense small particles, while the formation of rubber in the vacuoles is related to the filamentous structure. In the rubber tree *Hevea brasiliensis*, there are different views regarding the synthesis of rubber. Southorn [5] proposed that rubber particles originate from the endoplasmic reticulum; on the contrary, some scholars proposed that rubber particles originate in the form of particles from the cytosol [41]. Hébant [42] and Wu and Hao [8] proposed that, in *Hevea brasiliensis*, the origin of small rubber particles has no morphological correlation with other organelles, and these particles are freely produced. In this study, rubber precursors including membrane-like structures, irregular flocculent substances, or black osmiophilic mass were often observed in cytosol close to degenerated plastids, mitochondria, and nucleus, or appeared in the vacuoles, which indicates no morphological correlation with specific organelles. We postulated a free producing process of rubber precursors in *Decaisnea insignis*, similar to the viewpoint proposed by Hebant [42] and Wu and Hao [8] for *Hevea brasiliensis.*

### 4.3. Vacuole-Mediated Autophagy during Rubber Biosynthesis

In *Decaisnea insignis*, rubber precursors originate from the degenerated cytoplasm, and later, the volume increases during the late sunken stage or expanding stage, when plenty of rubber precursors or rubber particles are engulfed by the vacuoles, indicating a vacuole-mediated autophagy process [43], and detailed study is still needed. A similar conclusion was drawn by Backhaus [2], who proposed that the accumulation of rubber particles in Parthenium plants is a vacuole-mediated autophagic event, that is, rubber originating from the cytosol is temporarily stored in the cytosol and then accumulated in the central large vacuole.

### 4.4. Exploring the Cytological Relationship between Rubber Accumulation and PCD

Rubber biosynthesis in plants is a fascinating biochemical system. During the earliest stage, a small number of membrane-like structures or a little amount of black osmiophilic mass, namely, rubber precursors, could be observed in the cytoplasm as ultrastructures, while the secretory epidermal cells were characterized by their small volume with dense cytoplasm and evident nuclei. In our study, at the cellular level, rubber precursors first appeared in the cytoplasm as membrane-like structures or small irregular particles, and their volume increased gradually in *Decaisnea insignis*, which was also reported in *Hevea brasiliensis* [2]. Unlike the earliest stage, more irregular rubber precursors were randomly observed in the disorganized cytoplasm or close to the degraded plastids, mitochondria, endoplasmic reticulum, and nucleus or in the vacuoles in sunken stage. Meanwhile, the deformed nucleus with condensed chromatin was further confirmed with the TUNEL-positive reaction of the secretory epidermal cells. Furthermore, the complete plasma membrane and vacuolar membrane and the degenerated organelles observed during the expanding stage could almost not be detected at the mature stage, and the secretory epidermal cells were characterized by plenty of rubber particles. In mature *Hevea*, a previous study had reported that the numbers of mitochondria or nuclei could not be detected in the laticifers [2], which agrees with our study. Histologically and cytologically, in our study, the nucleus gradually degraded, while rubber particles accumulated to a high degree.

Recent studies have been conducted on the different types of plant glands, and it is suggested that PCD may be a common phenomenon in the development and senescence of plant glands, during which various secretions accumulate after the degradation of protoplasts and various organelles [13,14,15,17,18,19,20,21,44]. The ontogenesis of laticiferous canal formation in the fruits of *Decaisnea insignis* was studied, considering a process of PCD, during which rubber particles mainly accumulated after the degeneration of protoplasts, indicating a close relationship between rubber accumulation and PCD. It is suspected that PCD may play a vital role in rubber synthesis, which is characterized by rubber precursors produced in the degenerated cytoplasm and autophagy-mediated rubber accumulation in the vacuoles.

### 4.5. Molecular Insights into Rubber Biosynthesis and PCD

The rubber synthesis pathway is a typical isoprene synthesis pathway. In *Hevea brasiliensis*, the phytosomes are metabolized via the MVA route or the MEP pathway to synthesize isopentenyl diphosphates, the precursors for rubber production. Isopentenyl diphosphates are subsequently converted to GGPPS and FPP and polymerized by REF, HRT, and SRPP to create rubber particles in the cytosol [11]. In the current study, the genes, *DiIPP*, *DiFPP* and *DiGGPPS*, related to rubber synthesis in the laticiferous canals of *Decaisnea insignis* pericarp changed significantly, which were located in the synthesis pathway’s upstream, showing a clear tendency to decrease from the earliest stage (S1) to the expanding stage (S3) and returning to normal levels of expression at the mature stage (S4), while the midstream *DiREF* exhibited its maximum expression level during the sunken stage (S2) and displayed a pattern of high and then low, both of which suggested that the rubber precursors were essentially synthesized before the expanding stage (S3). However, *DiHRT*, which controlled the extension of isoprene to form rubber particles and was found downstream of the synthesis pathway, showed the greatest expression during the expanding stage (S3), indicating a crucial factor for the formation of rubber particles. These results are essentially in agreement with the ultrastructural evidence and statistical analyses of rubber precursor- and rubber particle-containing cells in our study.

In a previous study, laticiferous canal formation in the fruits of *D. fargesii* (*D. insignis*) was considered to occur via a programmed cell death process, during which rubber particles accumulated [12]. In this study, two PCD negative regulators, LSD1 and LOL2, together with two typical cysteine proteases, CEP1 and XCP1, were used to examine the PCD process in the laticiferous canals of *Decaisnea insignis* pericarp. CEP1 was discovered to play a direct role in the removal of cell contents during PCD in Arabidopsis [25], while XCP1 also served as a mechanism for autolysis following damage or planned cell death [26]. LSD1 zinc finger protein keeps track of a superoxide-dependent signal and regulates a pathway that adversely leads to plant cell death [27]. Similarly, LOL2 has a unique zinc finger motif that negatively regulates cell death and the immune system, and it shares this pattern with LSD1 [45]. These findings indicate that *DiCEP1* and *DiXCP1* are highly expressed as proteases during early developmental stages, whereas *DiLSD1* and *DiLOL2*, which act as negative regulatory transcription factors, are expressed at very low levels. Conversely, the expression of the two proteases decreases during the developmental process of laticiferous canals, while *DiLSD1* and *DiLOL2* expression increases. This outcome is in line with those of cellular investigations as well.

Notably, the linear correlation analysis of the qRT-PCR results (Figure S1) showed that there was a significant negative correlation (*p* < −0.85) between the PCD-related transcription factors, *DiLSD1* and *DiLOL2*, and the expression pattern of the natural rubber synthesis gene *DiHRT*, as well as a high positive correlation (*p* > 0.9) between the expression trends of *DiXCP1*, *DiCEP1,* and *DiGGPPS*.

## 5. Conclusions

**Figure 15:**
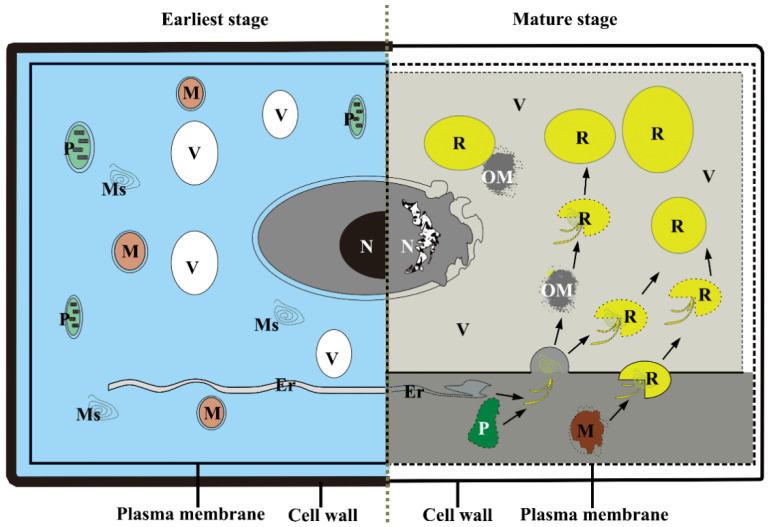
Schema for the relationship between rubber synthesis and PCD in the laticiferous canal of *Decaisnea insignis.* Substances metabolized by programmed cell death could likely be used for rubber synthesis in laticiferous canals of *Decaisnea insignis*. Er, endoplasmic reticulum; Ms, membrane-like structures; N, nucleus; OM, osmiophilic mass; P, plastid; R, rubber particle; and V, vacuole.

The findings of this study imply that PCD mediates the developmental process of laticiferous canals during *Decaisnea insignis* fruit development, and a strong correlation is detected between rubber synthesis and PCD in laticiferous canals (Supplementary Table S2), which raises the possibility that the two biological activities may be connected. In other words, it is speculated that substances metabolized by programmed cell death could likely be used during rubber synthesis to form laticiferous canals in *Decaisnea insignis*. Finally, a schema for the relationship between rubber synthesis and PCD in the laticiferous canals of *Decaisnea insignis* is presented (Figure 15).

## 6. Future Directions

Rubber biosynthesis in plants is a fascinating biochemical system, and its biosynthesis mechanism remains largely unknown, although a great number of studies have been conducted. In our study, a close relationship between rubber biosynthesis and PCD during the formation of laticiferous canals in *Decaisnea insignis* was observed, coupled with an autophagy-mediated process. Autophagy may play a key role in PCD-mediated rubber synthesis, the mechanism of which remains to be further studied, with the aim of not only providing new evidence for the production and accumulation of natural rubber, but also providing scientific basis for the regulation of secondary metabolites in plant secretory structures.

## Data Availability

Transcriptome sequencing data are available in the SRA database of National Center for Biotechnology Information (NCBI) under the accession number of PRJNA998538.

## Supplementary Materials

The following supporting information can be downloaded at: https://www.mdpi.com/article/10.3390/plants12193497/s1. Figure S1: Linear correlation analysis of qRT-PCR results of different genes; Supplementary Table S1: qRT-PCR primers; Supplementary Table S2: Cytological, genetic, and transcriptional changes in different developmental stages of laticiferous canal in *Decaisnea insignis* fruit.

## Abbreviation

GGPPS: geranylgeranyl pyrophosphate synthase; IPP: isopentenyl diphosphates; FPP: farnesyl pyrophosphate synthetase; REF: rubber elongation factor; HRT: Hevea brasiliensis cis prenyltransferases or rubber transferase; XCP1: xylem cysteine peptidase 1; CEP1: cysteine proteinases superfamily protein; RD21A: granulin repeat cysteine protease family protein cysteine proteinase precursor-like protein/dehydration stress-responsive gene (RD21); and LSD1: lesion simulating disease1.
