# Supplementary material for: Novel Mechanisms Underlying Rubber Accumulation and Programmed Cell Death in Laticiferous Canals of Decaisnea insignis Fruits: Cytological and Transcriptomic Analyses
Source: Plants (Basel). 2023 Oct 7;12(19):3497. doi: 10.3390/plants12193497 (PMC10575083; doi:10.3390/plants12193497)
Supplement: Supplementary file 1 [file plants-12-03497-s001.zip › Figure S1.pdf]

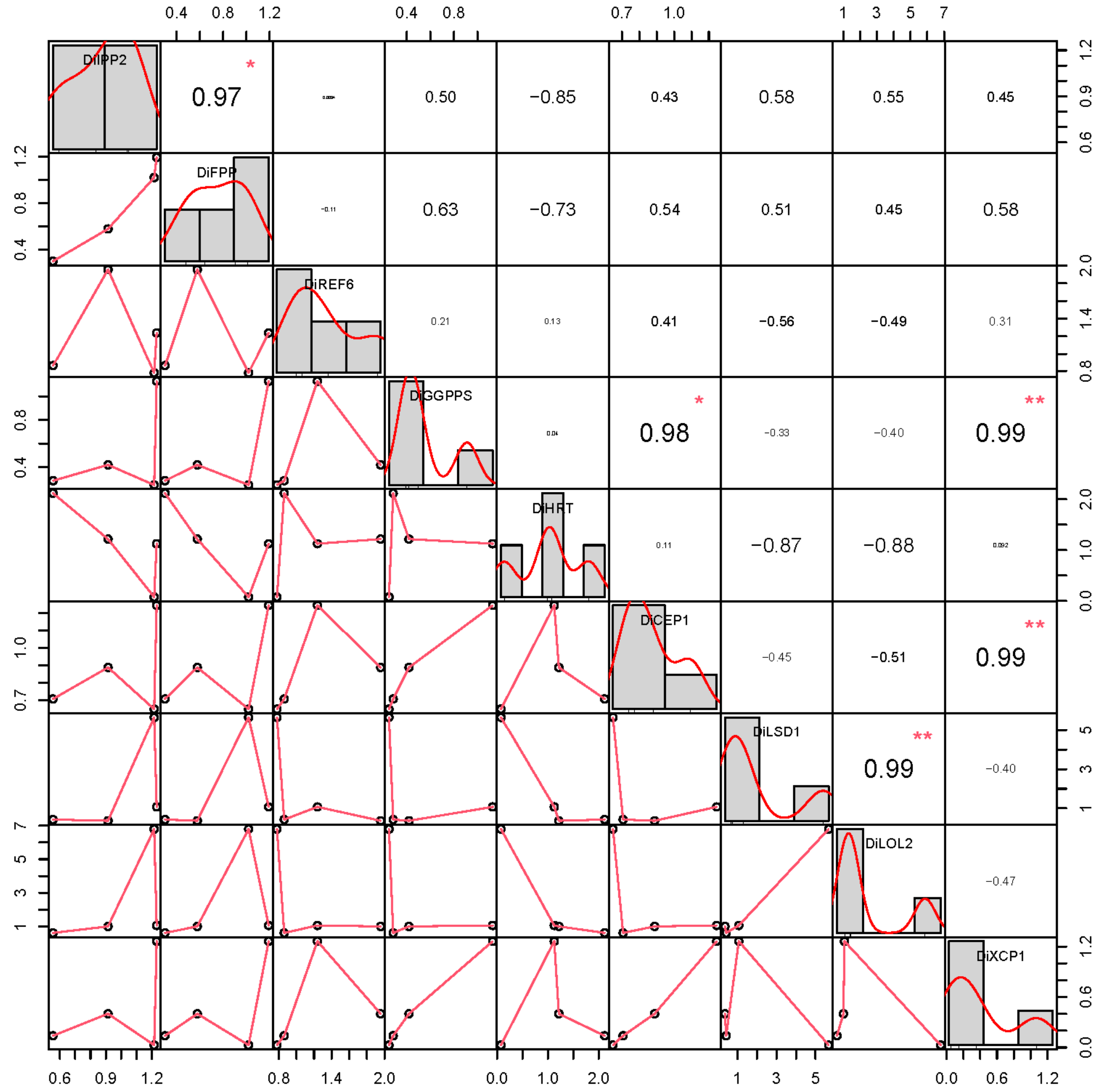

Figure S1: Linear correlation analysis of qRT-PCR results of different genes. The bar chart with the gene name indicates that the histogram of the frequency distribution of the qRT-PCR results of the gene; the cross position of different genes indicates the strength of the linear relationship between the two genes, the cross position in the lower left corner between different genes is the scatter plot of the qRT-PCR results of the two genes, and the cross position in the upper right portion indicates that, the magnitude of p-value of the qRT-PCR results between the two genes.
