# Supplementary material for: Novel Mechanisms Underlying Rubber Accumulation and Programmed Cell Death in Laticiferous Canals of Decaisnea insignis Fruits: Cytological and Transcriptomic Analyses
Source: Plants (Basel). 2023 Oct 7;12(19):3497. doi: 10.3390/plants12193497 (PMC10575083; doi:10.3390/plants12193497)
Supplement: Supplementary file 1 [file plants-12-03497-s001.zip › Supplementary Table S1.pdf]

**Supplementary Table S1 qRT-PCR Primers.**

| Primer name | Primer sequence（5’-3’） | Primer length (bp) |
|---|---|---|
| *DiAct1*-F | AGTTCTCCTCACCGAAGCAC | 20 |
| *DiAct1*-R | CCCTTCGTAGATGGGAACCG | 20 |
| *DiIPP2*-F | GCACATGCAGAAGCTATACGG | 22 |
| *DiIPP2*-R | TAATGCTCTCTCCGGTGCTC | 20 |
| *DiFPP*-F | TCGACTTCTTCACCACTGGC | 20 |
| *DiFPP*-R | AGGAGATGGATCGTCGGAGT | 20 |
| *DiREF6*-F | AGTTTCGAGGGCAAAAGGGT | 20 |
| *DiREF6*-R | TCTCTGGGCACTCCATACCA | 20 |
| *DiGGPPS*-F | TCGAGGTTCATCACGTCGTC | 20 |
| *DiGGPPS-R* | GAGATCTACTGGCGCGAGAC | 20 |
| *DiHRT1*-F | TAGGGAGTTTGGTGCGAAGG | 20 |
| *DiHRT1-R* | CACGTACTTCACCCCGAGTT | 20 |
| *DiLSD1*-F | TTGTGTGACAGCAGGAGCAT | 20 |
| *DiLSD1*-R | CTTGTGTGCAACGGGTGTAG | 20 |
| *DiLOL2*-F | CCGACAGGACAGCAATCCAT | 20 |
| *DiLOL2*-R | CCGACAGGACAGCAATCCAT | 20 |
| *DiXCP1*-F | TGCAAACTCATTCAACCCAAGC | 22 |
| *DiXCP1*-R | TGGGTTATTCACCGGACGAC | 20 |
| *DiCEP1*-F | CTGTTGCTAACCAGCCCGTA | 20 |
| *DiCEP1*-R | CCAGTTGGCTCCCCATGAAT | 20 |
