# Supplementary material for: Novel Mechanisms Underlying Rubber Accumulation and Programmed Cell Death in Laticiferous Canals of Decaisnea insignis Fruits: Cytological and Transcriptomic Analyses
Source: Plants (Basel). 2023 Oct 7;12(19):3497. doi: 10.3390/plants12193497 (PMC10575083; doi:10.3390/plants12193497)
Supplement: Supplementary file 1 [file plants-12-03497-s001.zip › Supplementary Table S2.pdf]

Supplementary Table S2 Cytological, genetic and transcriptional changes in different developmental stage of laticiferous canal in *Decaisnea insignis* fruit

Evants	Stage 1	Stage 2	Stage 3	Stage 4
Rubber accumulation	A little amount of brown irregular flocculent	Membrane structures and obvious osmiophilic mass in cytoplasm; vacuoles with increasing membrane structures; dark osmiophilic material and diffused flocculent material from the engulfed cytoplasm	A little amount of irregular flocculent and plenty of brown rubber particles	Full of round or elliptical rubber particles
	DiIPP2	↓	↓	↑
	DiFPP	↓	↓	↑
	DiREF6	-	↓	-
	DiHRT	↑	↑	↓
	DiGGPPS	-	↓	-
PCD characters	Relatively small size; dense cytoplasm; large nucleus; and small vacuoles	Thinning of the cell walls; the cytoplasm with more condensed feature; DNA fragmentation and TUNEL positive nuclei, misshapen nucleus with condensed chromatin; plastids with condensed matrix and indistinct thylakoids; misshapen mitochondria with disorganized membrane	Volume of secretory epidermal cells enlarged with a fivefold to eightfold increase; plastids with degenerated thylacoid system, mitochondria, and misshapen nucleus in vacuole; the nucleus enveloped by the rubber granule	The secretory epidermal cell walls with a thinner shape and even lysis of some parts; tonoplast rupture; plasma membrane collapse
	DiCEP1	↓	↓	-
	DiXCP1	-	↓	-
	DiLSD1	↓	↓	↑
	DiLOL2	-	↑	↑

Note: The arrows indicate trends at gene expression levels based on transcript FPKM values: a "↑↓" arrow indicates that the gene is significantly (p < 0.05) up or down regulated from the previous stage, and a "-" indicates that the gene is not significantly different from the previous stage.
